# Genome Sequence of the Virulent Model Herpes Simplex Virus 1 Strain McKrae Demonstrates the Presence of at Least Two Widely Used Variant Strains

**DOI:** 10.1128/MRA.01146-19

**Published:** 2021-03-25

**Authors:** Daniel W. Renner, Lance Parsons, Jacob T. Shreve, Esteban A. Engel, Chad V. Kuny, Lynn Enquist, Donna Neumann, Colleen Mangold, Moriah L. Szpara

**Affiliations:** a Department of Biology, Pennsylvania State University, University Park, Pennsylvania, USA; b Lewis-Sigler Institute for Integrative Genomics, Princeton University, Princeton, New Jersey, USA; c Department of Biochemistry and Molecular Biology, Center for Infectious Disease Dynamics, Huck Institutes of the Life Sciences, Pennsylvania State University, University Park, Pennsylvania, USA; d Princeton Neuroscience Institute, Princeton University, Princeton, New Jersey, USA; e Department of Molecular Biology, Princeton University, Princeton, New Jersey, USA; f Department of Ophthalmology and Visual Sciences, University of Wisconsin School of Medicine and Public Health, Madison, Wisconsin, USA; g Department of Entomology, Pennsylvania State University, University Park, Pennsylvania, USA; DOE Joint Genome Institute

## Abstract

Herpes simplex virus 1 (HSV-1) strain McKrae was isolated in 1965 and has been utilized by many laboratories. Three HSV-1 strain McKrae stocks have been sequenced previously, revealing discrepancies in key genes. We sequenced the genome of HSV-1 strain McKrae from the laboratory of James M. Hill to better understand the genetic differences between isolates.

## Abstract (precis)

Herpes simplex virus 1 (HSV-1) strain McKrae was isolated in 1965 and has been utilized by many laboratories. Three HSV-1 strain McKrae stocks have been sequenced previously, revealing discrepancies in key genes. We sequenced the genome of HSV-1 strain McKrae from James Hill, to better understand the genetic differences between isolates.

## ANNOUNCEMENT

Herpes simplex virus 1 (HSV-1, or human herpesvirus 1, HHV-1) is a neurotropic and neuroinvasive double-stranded DNA virus of the *Herpesviridae* family (genus *Simplexvirus*, subfamily *Alphaherpesvirinae*), which infects humans. HSV-1 strains have been shown experimentally to exhibit wide variation in both virulence and pathogenic phenotypes (1, 2). The HSV-1 strain McKrae was originally isolated from a human sample and has been passaged through multiple cell types and species (3). McKrae is one of the most highly virulent model strains of HSV-1, demonstrating a high reactivation frequency and neurovirulence in animal models (1, 4–6). There are currently three variant strains of McKrae with genome sequences in GenBank (submitted under accession no. JX142173.1 [7], JQ730035.1 [8], and MN136524 [9]). The differences between these published genome sequences—for instance, the putative JQ730035.1 truncations of the UL56 and UL36 open reading frames (ORFs) and an extension of the US10 ORF (see reference 8 for details)—led us to sequence the genome of HSV-1 McKrae from the laboratory of James M. Hill (located at Louisiana State University Health Sciences Center until his death in 2013). Hill often utilized stocks of HSV-1 McKrae in his comparisons of HSV-1 virulence in mice and rabbits, and he freely shared these resources with other labs (1, 10, 11).

We isolated viral nucleocapsid DNA from HSV-1 McKrae-infected Vero (African green monkey kidney) cells, using published methods (12, 13). Cells infected at a high multiplicity of infection (MOI = 10) were scraped, host membranes were disrupted via Freon extraction, and viral capsids were separated from cellular debris by pelleting through a density gradient. After detergent and proteinase K lysis of capsids, the viral nucleocapsid DNA was further purified using phenol-chloroform extraction, followed by ethanol precipitation of the viral genomic DNA. We followed the manufacturer’s protocols (Illumina TruSeq DNA) to produce a barcoded library of 500-bp fragments and to obtain 16.3 million 100-bp, paired-end sequence reads (Illumina HiSeq 2000 with v2 chemistry).

The viral genome assembly process has been previously described (14) and is summarized here, with full installation and usage instructions at http://virga.readthedocs.org/. All scripts were used with default parameters unless stated otherwise. For quality control prior to genome assembly, we first trimmed any library adapter sequences, sequencing artifacts, and low-quality bases using FastX-Toolkit and Trimmomatic (15). Then, Bowtie 2 was used to map and remove any host-derived DNA sequences (using the rhesus macaque genome sequence as a proxy for Vero cell DNA) (14, 16). After quality control and host-contaminant removal, 11.7 million paired-end sequence reads were used as the input for multiple SSAKE v3.8 *de novo* assemblies (using eight pairs of overlap and trimming parameters, 16-0, 19-0, 20-0, 29-0, 16-4, 19-4, 20-4, and 29-4). These SSAKE contigs were combined into larger sequence blocks using Celera v8.1 (14). We then used Mugsy v2.3 to align these contigs to the HSV-1 reference genome sequence (GenBank accession no. JN555585) and stitched them into a consensus genome sequence using a custom script, maf_net.py (https://bitbucket.org/szparalab/virga/src/master/), and GapFiller v1.10 (insert_deviation 0.2) (14). Finally, we used a genome-wide alignment (ClustalW2 v2.1) of the new consensus genome sequence with the HSV-1 reference genome sequence (JN555585) as a template to transfer annotations, using the custom script compare_genomes.py (https://bitbucket.org/szparalab/virga/src/master/) (14). This new HSV-1 McKrae genome sequence (KT425107) is 149,310 bp, with 6 gaps in comparison to the HSV-1 reference genome sequence (JN555585). The new McKrae genome sequence has 67.8% GC content and an average sequence depth of coverage exceeding 1,000× (with 97.5% of the genome at ≥100× depth).

**FIG 1:**
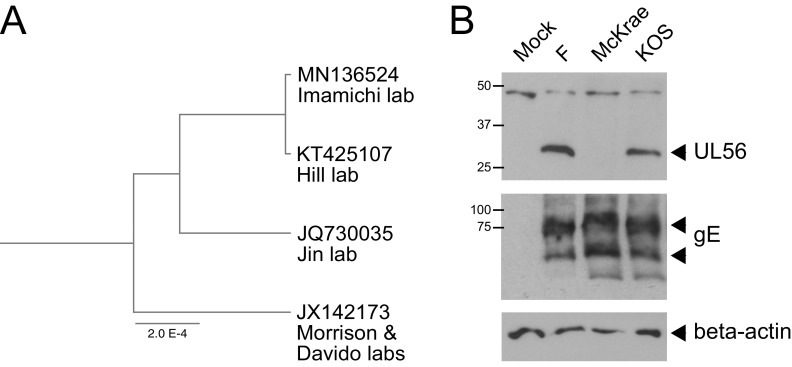
Strain McKrae genomes are closely related but do not all express intact UL56 protein. (A) A UPGMA tree built based on a whole-genome alignment of four strain McKrae genome sequences demonstrates their high relatedness (excluding gaps). (B) However, the UL56 gene in the Hill lab McKrae and two other published McKrae genome sequences has a frameshift and early truncation, which results in a lack of UL56 protein. Western blots of protein lysates are shown from epithelial Vero (monkey kidney) cells at 6 hours postinfection (hpi), including a mock infection, as well as HSV-1 strains F, McKrae (Hill lab; GenBank accession no. KT425107), and KOS. Viral glycoprotein E (gE) demonstrates infection for all three strains, and beta-actin serves as a loading control. The conditions for Vero cell infection and Western blotting were the same as those for our previously published studies (21, 22). Antibody sources: mouse anti-beta-actin, Sigma-Aldrich; mouse anti-gE, kindly provided by Harvey Friedman; rabbit anti-UL56 antibody, generated by Esteban Engel.

We performed genome alignments between this McKrae genome sequence (KT425107) and those previously published by the Jin laboratory (JQ730035) (8), the Morrison and Davido laboratories (JX142173) (7), and the Imamichi laboratory (MN136524) (9), using MAFFT v7.475 (FFT-NS-2 with default parameters; https://mafft.cbrc.jp/alignment/server/). We generated a tree from all gap-free sites in the full-genome sequence alignment (145,746 bases), using the UPGMA method (Jukes-Cantor model; Geneious v11.1.5). This indicated that three of the McKrae genomes are very closely related, with JX142173 from the Morrison and Davido laboratories separated by a small margin (Fig. 1A). With the exception of the UL56 variant noted below, none of the ORF truncations or extensions described in the prior publication on McKrae strain JQ730035 (8) were detected in this new McKrae genome.

One of the major differences between the prior published variants of HSV-1 McKrae was in the UL56 gene—it has a truncated open reading frame (ORF) in JQ730035 and MN136524 and an intact ORF similar to other HSV-1 strains in JX142173 (17). We found an identical truncation in the Hill lab McKrae strain, KT425107 (the current genome sequence), and the previously described McKrae strains JQ730035 and MN136524. In contrast, the UL56 ORF in the Morrison and Davido laboratories’ McKrae strain JX142173 is full length and appears to be intact (7). The nonintact UL56 genes have a frameshift (due to an extra G nucleotide) after amino acid 97, an altered amino acid tail, and an early stop codon at amino acid 181 (versus a full-length protein of 234 amino acids in other strains). We have verified the absence of the UL56 protein product in the Hill lab McKrae strain KT425107 (Fig. 1B). While an intact UL56 gene has been shown to be required for HSV-1 virulence *in vivo* (18–20), all strains used in those prior studies are less virulent in animal models than HSV-1 McKrae (1, 2, 10, 11). These data raise the question of whether or not the UL56 protein is required in the HSV-1 McKrae genetic background to achieve full virulence in animal models. We encourage other researchers to evaluate the status of the UL56 gene product in their own stocks of this model virulent HSV-1 strain to help address this question.

### Data availability.

The HSV-1 McKrae genome sequence has been deposited at GenBank under the accession no. KT425107. The raw reads have been submitted to the SRA as accession no. SRR13801763.
